# How do the experiences and beliefs of adults and children with heterozygous familial hypercholesterolaemia influence their adherence to treatment? A systematic review of qualitative evidence protocol

**DOI:** 10.1186/s13643-018-0793-7

**Published:** 2018-08-16

**Authors:** Fiona J. Kinnear, Rachel Perry, Aidan Searle, Julian P. Hamilton-Shield, Fiona E. Lithander

**Affiliations:** 1 NIHR Bristol Biomedical Research Centre (Nutrition Theme), University Hospitals Bristol NHS Foundation Trust, Bristol, UK; 2 University of Bristol, Bristol, UK

**Keywords:** Familial hypercholesterolaemia, Children, Adolescents, Diet, Physical activity, Cholesterol, Systematic review, Qualitative research, Treatment adherence, Cardiovascular heart disease

## Abstract

**Background:**

Heterozygous familial hypercholesterolaemia (FH) is a genetic disorder characterised by elevated levels of low density lipoprotein (LDL) cholesterol from birth, estimated to affect 1 in 250 of the UK population. Left untreated, FH substantially increases an individual’s risk of premature coronary heart disease (CHD) and associated mortality. This risk can be minimised with timely diagnosis and successful treatment with medication and lifestyle changes, as advocated in national and international guidelines. Despite these recommendations, the limited research available suggests adherence to treatment may be sub-optimal. This review will identify and synthesise the available qualitative research regarding the experiences and beliefs of adults and children with FH in relation to their condition and its treatment, and the influence of these upon treatment adherence.

**Methods:**

The following electronic databases will be searched from their inception: Cochrane library, MEDLINE, Embase, PsycINFO (via OVID) and CINAHL. Studies available in English and reporting primary qualitative data will be included. Database searching will be supplemented with searches in relevant specialist websites. The references of identified papers will also be hand searched. Two reviewers will independently screen titles and abstracts of identified studies, with full texts of potentially relevant papers retrieved for review against pre-defined inclusion and exclusion criteria. The Critical Appraisal Skills Programme (CASP) Qualitative Research checklist will be used to assess quality of the included studies, and the results will be taken into consideration when reporting the findings. A data extraction tool will be created for use in this review to extract study findings relevant to the review questions. A thematic synthesis approach will be taken to analyse the results.

**Discussion:**

Adherence to treatment recommendations is crucial for the successful management of FH and subsequent decrease in risk of CHD later in life. Common identified themes could provide an understanding of the beliefs and experiences which influence adherence to treatment recommendations and provide an insight into perceived barriers and facilitators. The findings are intended to be used in the development of future interventions or guidelines regarding treatment of children and adults with FH.

**Systematic review registration:**

PROSPERO registration number: CRD42018085946

**Electronic supplementary material:**

The online version of this article (10.1186/s13643-018-0793-7) contains supplementary material, which is available to authorized users.

## Background

Familial hypercholesterolaemia (FH) is an autosomal dominant hereditary disorder, characterised by markedly elevated levels of low-density lipoprotein (LDL) cholesterol from birth [1]. This review will focus on heterozygous FH (henceforth to be known as FH) which has been estimated to affect 1 in 250 of the UK population [2] and substantially increases risk of premature coronary heart disease (CHD) and associated mortality [3, 4]. However, this risk can be reduced with appropriate lifelong treatment [5]. UK and international guidance recommends that individuals with FH should commence life-long lipid-lowering drug therapy from 10 years of age, alongside the adoption of lifestyle advice which aims to encourage maintenance of a healthy weight, smoking avoidance, participation in regular physical activity (PA), limited alcohol consumption and adherence to a healthy, balanced diet [6, 7].

There are very little data regarding medication and lifestyle advice provision and adherence in the FH population [8]. From the available evidence, it appears that healthcare professionals are following the guidelines with the majority of adults [9], but treatment provided to children is inconsistent [4, 10]. While adherence to medication in adults appears to be high [11–13], a Dutch questionnaire survey found that only 49% of adults with FH adhere to dietary and PA recommendations [11]. Similarly, the adoption of lifestyle recommendations has been reported to be low in children [10].

Qualitative research can provide an insight into an individual’s understanding, perceptions and beliefs of their condition and its management. This can enable the development of an understanding of how and why individuals’ display certain behaviours and an awareness of factors influencing their decisions to adhere to treatment [14]. Illness perceptions and beliefs about treatment have been found to influence treatment compliance in adults with chronic conditions such as asthma [15, 16], epilepsy [17] and hypertension [18]. Specifically, necessity and concern beliefs have been found to be modifiers of treatment adherence across a range of health conditions [19]. Similar findings have been found for children and adolescents with asthma [20], cystic fibrosis [21] and type 1 diabetes [22]. Within the FH population, adherence to treatment has been found to be influenced by several factors including knowledge of FH [23], perception of risk [24], beliefs about influence of treatment upon long-term health [23] and personal experiences such as family history of cardiovascular disease [25]. Furthermore, barriers to adhering to treatment have been highlighted including a lack of motivation [23], social acceptance [24] and concerns regarding medication [26]. From the limited research conducted in young adults with FH, experiences of family history of disease has been found to influence decisions regarding treatment [25] and lack of credible information, motivation and symptoms highlighted as barriers to adhering to treatment [25, 27].

Results from these individual studies provide useful insights but are restricted by their small sample sizes and are subject to bias resulting from the researcher’s interpretation of the participants’ views, and the quotes they select to present in the findings. By conducting a systematic review of qualitative studies, a wider range of patient perceptions and experiences can be examined which allows for common themes to be identified and explored in more depth [28]. Furthermore, it could provide awareness of common enablers and barriers to treatment adherence within the patient population group. These findings would be useful in the development of future interventions or recommendations for the FH population. To date, one systematic review of qualitative research in the FH population has been carried out [29]; however, it aimed to answer questions regarding the influence of screening approaches on diagnostic rates. Thus far, no synthesis of qualitative data regarding the experiences and beliefs of individuals with FH in relation to their condition, its treatment and the influence of these upon treatment adherence has been carried out.

### Research objective

This systematic review aims to identify and synthesise the available qualitative evidence regarding the experiences and beliefs of adults and children with FH in relation to their condition, its treatment and the influence of these upon treatment adherence. This review also seeks to identify any perceived enablers and barriers to treatment adherence. The findings are intended to be used in the development of future interventions or guidelines targeting medication and lifestyle behaviours in children and adults with FH.

### Research questions

In adults and children with FH:

- What are the experiences and beliefs of individuals’ in relation to their condition, its associated morbidity and mortality risk and treatment?
- How do these experiences and beliefs influence individuals’ adherence to pharmacological and lifestyle treatment recommendations?
- Are there any enablers and/or barriers to adhere to pharmacological and lifestyle treatment recommendations?
- Do the findings differ between adults and children?

## Methods

Where appropriate for qualitative reviews, the reporting of this protocol is in accordance with the ‘Preferred Reporting Items for Systematic Review and Meta-Analysis Protocols (PRISMA-P) 2015 checklist’ [30]. A completed checklist for this review protocol is available in Additional file 1. This protocol will also be reported in accordance with the enhancing transparency in reporting the synthesis of qualitative research (ENTREQ) statement [31].

### Eligibility criteria

Papers reporting studies that meet the following criteria will be included in the analysis.

#### Participants

The participants include individuals aged ≥ 10 years with clinically diagnosed FH. In this review, a genetic diagnosis of FH is not required for inclusion if the study clearly states that the study population has all received a clinical diagnosis. The age of 10 years was used as a cutoff as this is age that children with FH are currently advised to be considered for treatment with statins and to attend subsequent follow-up appointments in a FH specialist clinic in the UK [7]. Individuals with a history of cardiovascular heart disease will be included. Individuals with a diagnosis of homozygous FH will be excluded.

#### Phenomena of interest

This review will consider any studies that investigate the experiences and beliefs of individuals with clinically diagnosed FH in relation to their condition, its associated morbidity and mortality risk and recommended pharmacological and lifestyle change treatment. Studies focusing on specific elements of FH which are unrelated to the research objectives of this report, such as attitudes towards genetic testing, will be included if the study also includes findings relevant to this review which can be independently extracted. Studies in which the focus is upon the perceptions and experiences of the family members, for example, parents of children and adolescents with FH, will also be included.

#### Type of studies

This review aims to explore the experiences and beliefs of individuals which are most appropriately done through analysis of qualitative data, and as such, only qualitative studies that report primary data will be included. For the purpose of this review, qualitative research refers to studies in which widely recognised qualitative methods of data collection and data analysis have been used. This includes, but is not limited to, data collection methods such as focus groups and interviews, and data analyses methods including narrative analysis and grounded theory [32]. Only studies in which the full text is available in English will be eligible for inclusion. This decision was taken due to the potential problems in interpreting and translating qualitative data in another language by the research team and lack of resource to carry this out. Mixed method studies, in which both quantitative and qualitative methods were used, will be included if it is possible to extract only the qualitative data. Quantitative studies, commentaries or reviews on the subject will be excluded. Studies which exclusively collected information through self-reported questionnaires or researcher administered surveys will be excluded as this does not allow for an in-depth understanding of individuals’ beliefs.

#### Intervention/exposure

In this review, treatment is defined as any action taken by individuals with FH in an attempt to manage their condition as a result of their own knowledge of the condition, advice given to them by a healthcare professional or from other family members. This includes, but is not limited to, behaviours relating to medication, weight management, smoking, diet and physical activity. Variations in the length of time since diagnosis and the mode of delivery, intensity and content of treatment advice which individuals will have received are expected; however, no exclusions will be made based on this criteria.

#### Setting

Studies will be included regardless of the country in which they were conducted and the setting in which the qualitative data were collected from individuals.

### Search strategy

The search will be systematic, pre-planned and comprehensive in order to find all available studies, as recommended for thematic synthesis [31, 33]. The search strategy will be developed by two reviewers (FK, RP) with reference to the available guidance and recommendations [31, 34, 35], as well as consultation with an information specialist with previous experience in designing qualitative search strategies. It will be carried out by one reviewer (RP).

#### Electronic searches

Literature searches will be undertaken in the following databases for published articles: MEDLINE, Embase, PsycINFO (via OVID), Cochrane library and CINAHL. All will be searched from the inception of the database to present. Search terms relating to the population will be combined with a validated search filter [36] containing terms relating to qualitative methods and methodology. The search will use a combination of subject headings (e.g. MeSH) and text words. A draft of the search strategy intended for use in MEDLINE is available in Additional file 2. The same search strategy will be used in the other listed databases, with appropriate subject headings and database-specific modifications to syntax and qualitative search filter applied.

#### Searching other resources

The reference lists of all included studies will be hand searched for additional studies. As qualitative research is often found in grey literature such as conference abstracts and theses [31], the OpenGrey database will be searched. Relevant specialist websites, including charities and government health departments with a focus in lipid, cardiac or genetic disorders will also be searched, e.g. American Heart Association, the international FH Foundation, the British Heart Foundation and the Simon Broome Register. Authors of any identified abstracts will be contacted to establish if full text is available. Experts in the area will be contacted to enquire about the existence of any unpublished work.

### Data management

The search results will be managed using Endnote reference management software. The results from the database searches will be imported into an Endnote library, with duplicates removed during the screening of the data.

### Selection of studies

Two reviewers (FK, RP) will independently screen the titles and abstracts of all retrieved records for inclusion. Full-text copies of potentially relevant studies will be retrieved for in-depth review against the inclusion criteria. The full-text articles will be screened independently by two reviewers (FK, JHS). Explanations for exclusion will be recorded at the full-text stage. A Preferred Reporting Items for Systematic Review and Meta-Analysis (PRISMA) flow chart will be produced to illustrate the different stages of study selection. Full-text articles which are judged to meet inclusion criteria will be taken forward to appraisal and data extraction stages.

### Data extraction

Firstly, the contextual and methodological information of the included studies will be extracted into a study table designed for this review, as recommended [34]. Information on the following will be extracted:

- Publication information: author, year of publication, country of study
- Sample characteristics: number of participants, demographics, FH diagnostic criteria
- Study design: methodological approach, conceptual bases underlying study, analysis method, setting
- Study objectives or aims

Secondly, in line with Thomas and Harden’s approach [33], study findings (data) will be extracted using a tool designed specifically for this review. All text and quotations reported in the ‘results/findings’, ‘discussion’ and ‘conclusion’ sections of papers, as well as in any supplemental files provided by the authors, will be considered for extraction. This inclusive approach to data extraction has been taken to minimise risk of missing findings which may be of potential value to the synthesis, as it is recognised that authors may report useful data out with the ‘findings’ section [34]. Any data judged to be relevant to the research questions of this review will be extracted. This data will take the form of first-order constructs (participants’ quotes and author summaries of quotes) and second-order constructs (author’s interpretations).

Both stages of data extraction will be carried out independently by two reviewers (FK, AS), with any disagreement resolved by discussion until consensus is reached, with a third reviewer (FL) consulted if required. The data extraction tool will be piloted on the first five studies, after which its suitability for use will be discussed and any required amendments made before continuation of data extraction on the remaining studies. Microsoft Excel or NVivo software will be used to manage the extracted data.

### Critical appraisal of study quality

The Critical Appraisal Skills Programme (CASP) Qualitative Research Checklist [37] will be used to assess the methodological validity of included studies. This will be carried out by two reviewers (FK, AS), with any disagreements discussed with a third reviewer (RP or FL). This tool was chosen as it can be applied to qualitative research of various designs, is frequently used in the literature [31] and is a recommended tool [34].

To avoid the possibility of excluding potentially valuable insights that can be generated from qualitative studies assessed to be inadequately designed or reported [28], the results of the appraisal will not be used to exclude any study. In their worked example of thematic synthesis methodology, Thomas and Harden [33] chose not to exclude studies as there is no widely accepted and empirically tested method for excluding studies based on their quality. However, it is recommended that the quality of included studies should be critically analysed in the subsequent data synthesis and be considered when drawing conclusions in systematic reviews of qualitative research [31]. Therefore, the appraisal findings will be presented alongside the findings of the review. Furthermore, after synthesis, the relative contributions of studies to the derived analytic themes and any recommendations which are made based on these will be explored in relation to their evaluated quality.

### Data synthesis

There are many recognised methods for qualitative synthesis, with no gold standard method recommended for use [38]. Instead, it is advised that the choice of method should be informed by the type and scope of review question and the content of the available literature [34]. Thematic synthesis is a method developed by Thomas and Harden [33] to answer questions addressing the suitability and acceptability of health promotion and public health interventions. The benefit of including the experiences and perceptions of the individuals being targeted in the interventions when assessing the effectiveness of such interventions was recognised, and thematic synthesis was developed to analyse and understand these qualitative data. The comparable research aims of this review, and its recommendation for use in qualitative systematic reviews [34], led to the decision to choose thematic synthesis.

Thematic synthesis is comprised of three main stages—line by line coding, development of descriptive themes and generation of new analytical themes from consideration of all the available data [33]. Line by line analysis will be carried out on the extracted data, with each first or second order construct identified being assigned codes. These codes will be inductively created in response to the findings uncovered. This will be carried out independently by two reviewers (FK, AS) with a third reviewer (FL) consulted to resolve any disparities. The two reviewers (FK, AS) will then work in collaboration to develop initial descriptive themes and categories based upon the raw data that closely reflect the aggregative findings of the included studies. Lastly, interpretative analytical themes based upon these aggregative findings will be generated where possible by the two reviewers. This stage seeks to go beyond the original findings of the studies to generate additional understanding and aims to produce recommendations for future interventions.

## Discussion

Adherence to treatment recommendations is crucial for the successful management of FH and subsequent reduction in risk of CHD later in life. This review will summarise the available qualitative evidence regarding the experiences and beliefs of adults and children with FH in relation to their condition and its treatment. Common identified themes could provide a greater understanding of the beliefs and experiences which influence the extent to which individuals follow current treatment recommendations and provide an insight into perceived barriers and facilitators to adherence. The findings are intended to be used in the development of future interventions or guidelines targeting medication and lifestyle behaviours in children and adults with FH.

## Additional files

Additional file 1: PRISMA-P (Preferred Reporting Terms for Systematic Review and Meta-analysis Protocol(s) 2015 checklist: recommended items to address in a systematic review protocol. PRISMA (Preferred Reporting Terms for Systematic Review and Meta-Analysis) is an evidence-based minimum set of terms for reporting in systematic reviews and meta-analyses. (DOCX 29 kb)

Additional file 2: Draft MEDLINE search strategy. (DOCX 14 kb)

## Abbreviations

CASP: Critical Appraisal Skills Programme
CHD: Cardiovascular heart disease
ENTREQ: Enhancing transparency in reporting the synthesis of qualitative research
FH: Familial hypercholesterolaemia
LDL: Low-density lipoprotein
MeSH: Medical Subject Headings
PA: Physical activity
PRISMA: Preferred Reporting Items for Systematic review and Meta-Analysis
PRISMA-P: Preferred Reporting Items for Systematic review and Meta-Analysis Protocols
