# Supplementary material for: How do the experiences and beliefs of adults and children with heterozygous familial hypercholesterolaemia influence their adherence to treatment? A systematic review of qualitative evidence protocol
Source: Syst Rev. 2018 Aug 16;7:120. doi: 10.1186/s13643-018-0793-7 (PMC6094562; doi:10.1186/s13643-018-0793-7)
Supplement: Supplementary file 2 — Draft MEDLINE search strategy. (DOCX 14 kb) [file 13643_2018_793_MOESM2_ESM.docx]

**Additional File 2: Draft MEDLINE search strategy**

1. (familial adj1 hypercholesterolemia).ti,ab, kf.

2. (familial adj1 hypercholesterolaemia).ti,ab, kf.

3. (inherit* adj1 high adj1 cholesterol).ti,ab, kf.

4. *Hypercholesterolemia/ge [Genetics]

5. 1 or 2 or 3 or 4

6. interview*.ti,ab.

7. exp. Interviews/

8. experience*.tw.

9. qualitative.ti,ab.

10. 6 or 7 or 8 or 9

11. 5 and 10
